# Payment for Notification

**Published:** 1898-04-30

**Authors:** 


### Editor's Letter-Box.

### PAYMENT FOR NOTIFICATION.

Dr. A. Newsholme, Medical Officer of Health for Brighton, writes: The remarks in The Hospital for the 16th inst., under the above head, do not completely represent my views and proposals. In the paper in *Public Health*, on which your comments are evidently based, I refer for details to an earlier paper in the "Journal of the Royal Statistical Society" (Vol. LIX., Part I., March, 1896), on "A National System of Notification and Registration of Sickness." I ask you, therefore, to publish the following extracts from the two papers in question.

Extract from "Public Health," April, 1898.

"Invidious Position of 'Public' Practice.—I have no hesitation in stating, in answer to question 7, that the notification forms should be postally prepaid, especially as in answer to question 3, I belong to the unpopular but, I believe, increasing minority, who believe that the fee for notification should be one shilling for all cases, and not simply for those occurring in the course of 'public practice.' How did such a distinction ever come to be made? If it was based on the assumption that the visiting medical officer of the poor law or of public dispensaries are likely to have a larger number of cases to notify than other medical practitioners, and that to pay 2s. 6d. would be too expensive, the former have a serious grievance in the invidious position they are thus made to occupy, a grievance which is not diminished by the considerations that they are the practitioners who can least afford to have their fees reduced, and that in the circumstances in which they commonly have to diagnose infectious diseases the difficulty is greater than in private practice. If anyone should receive a fee of 2s. 6d. it is the poor law medical officer, or the dispensary doctor, who is at present under the temptation, in the stress of hurried work, to diagnose infectious diseases after insufficient examination and inquiry."

Extract from the Statistical Society's Journal, March, 1896.

"In accordance with general principles, the State has no right to require work from private members of the community without giving remuneration for this work. On the other hand, the State has always required certain work from its citizens without such remuneration. Every ratepayer is liable to be called upon to act on a jury, often to the serious detriment of his private business, medical men being exempted from this duty. Further, the State has already required duties analogous to notification of sickness from medical practitioners. They must certify the cause of death of patients attended by them; and they must supply certificates of vaccination for children successfully vaccinated by them, both without remuneration. We shall shortly see that in none of the other countries to be mentioned, in which an elaborate system of notification is enforced, is any payment made for the information furnished by the medical practitioner. In these countries there are more rigorous regulations protecting qualified practitioners from the unscrupulous competition of unqualified persons. In this country such protection is most imperfect. If it were determined to extend the notification of sickness without any corresponding (if any) increase of expense for fees for notification, this should be accompanied by legislation giving medical practitioners that protection in the discharge of their skilled work which is required. We believe that if an enactment strictly prohibiting the practice of medicine by unqualified persons, and at the same time requiring weekly returns of all cases of a specified number of diseases were to be made, medical men would not seriously object to the gratuitous labour involved. Failing this, or as a preliminary to it, the expense of extended notification might be reduced by a modified system, weekly returns only, instead of immediate returns, being required in all except the more urgent infectious diseases."

The former extract states my views as to the action which is desirable when in the near future an amending Notification Bill is introduced; the latter extract my views as to the ultimate position which will, I hope be reached, which has already been reached in the chief European countries and in many of the United States.

⁂ The suggestion made in the second of these extracts is much more just to the medical profession than that on which we commented on the 16th inst. We quite believe that the medical profession would readily enough do much for the State if the State would, as Dr. Newsholme suggests, enforce "an enactment strictly prohibiting the practice of medicine by unqualified persons." We have not, however, the faintest expectation of seeing any such enactment passed. In the meantime, then, we think that the public should pay good and sufficient fees for the expert services which it requires for its own protection.—Ed. *T. H.*

